# A new species of *Gudeodiscus* Páll-Gergely, 2013 from China, with extraordinary conchological and anatomical features (Gastropoda, Pulmonata, Plectopylidae)

**DOI:** 10.3897/zookeys.564.6560

**Published:** 2016-02-16

**Authors:** Barna Páll-Gergely, Takahiro Asami

**Affiliations:** 1 Department of Biology, Shinshu University, Matsumoto 390-8621, Japan

**Keywords:** Taxonomy, systematics, anatomy, sympatric species, duplicated organ, plesiomorphic character

## Abstract

A new species of the Plectopylidae, *Gudeodiscus longiplica* is described from northern Guangxi Province, southern China. The shell, anatomical and radular characters are figured and described. This new species is characterized by long plicae on its parietal shell wall, which have not been observed in any other *Gudeodiscus* species. In contrast, the long parietal plicae are characteristic for the genera *Plectopylis* and *Chersaecia*, which mainly inhabit Thailand and Myanmar. These two genera are, however, only distantly related to the new species, as other characters (anatomy, protoconch sculpture, parietal plicae) suggest. The male portion of the genital structure of the new species is characterized by two separate penial caeca with different lengths, but similar in outer and inner structure. The relevance of this anatomical character is discussed. *Gudeodiscus longiplica* **sp. n.** occurs sympatrically with *Gudeodiscus soosi* Páll-Gergely, 2013. The anatomy and radula characters of the latter species are also described and figured.

## Introduction

The Plectopylidae are composed of flat-shelled terrestrial snail species which are characterized by a complex, internal armature structure. The armature is composed of plicae (horizontal structures) and lamellae (vertical structures) on both the palatal and parietal sides of the body whorl. These barriers are situated ¼ – ½ whorl behind the aperture, thus, usually not visible from the aperture. Instead, the palatal plicae can be seen through the semi-transparent shell wall, whereas small holes must be made in the shell at appropriate sites to examine the parietal plication. The morphology of these plicae and lamellae serve as primary diagnostic characters for species recognition and identification. In addition to these peculiar conchological features, unique traits in the anatomy of Plectopylidae have been reported, i.e. “disposable” calcareous, hook-like granules inside the penis lumen which are probably spent during mating (Páll-Gergely and Hunyadi 2013, Páll-Gergely et al. 2015a).

The most speciose genus in the Plectopylidae is *Gudeodiscus* Páll-Gergely, 2013, mainly distributed in the Chinese Guangxi Province and northern Vietnam, but some species have been reported from eastern Yunnan, southern Hunan and southern Guangdong provinces. More than half of the recorded 24 species of *Gudeodiscus* are known from only empty shells at this time (Páll-Gergely and Hunyadi 2013, Páll-Gergely and Asami 2014, Páll-Gergely et al. 2015a), including *Gudeodiscus soosi* Páll-Gergely, 2013 reported from three nearby localities in northern Guangxi. After *Gudeodiscus soosi* was described, we had the opportunity to examine four additional specimens (shells and ethanol-preserved bodies), from the original sample. Examination revealed that two specimens were *Gudeodiscus soosi*, but the other two were an undescribed species, which is described herein.

## Material and methods

Determination of number of shell whorls (precision to 0.25 whorl) follows Kerney and Cameron (1979: 13). Shells and radulae were directly observed without coating under a low vacuum SEM (Miniscope TM-1000, Hitachi High-Technologies, Tokyo). Individual buccal masses were removed and soaked in 2 M KOH solution for 5 h before extracting the radula, which was preserved in 70% ethanol. We use the terms “proximal” and “distal” in relation to the central of the body.

### Abbreviations

JUO
 Collection Jamen Uiriamu Otani (Koka, Japan) 


NHMUK
 The Natural History Museum (London, UK) 


OK
 Collection Kenji Ohara, Nishinomiya Shell Museum (Nishinomiya, Japan) 


PGB
 Collection Barna Páll-Gergely (Mosonmagyaróvár, Hungary) 


## Taxonomic descriptions

### Family Plectopylidae Möllendorff, 1898

#### Genus Gudeodiscus

Taxon classificationAnimaliaPulmonataPlectopylidae

Páll-Gergely, 2013

Gudeodiscus Páll-Gergely, In: Páll-Gergely & Hunyadi, Archiv für Molluskenkunde 142(1): 4, 8. 2013

##### Type species.

*Plectopylis phlyaria* Mabille, 1887, by original designation.

#### Subgenus Gudeodiscus

Taxon classificationAnimaliaPulmonataPlectopylidae

Gudeodiscus (Gudeodiscus) , — Páll-Gergely, et al., ZooKeys 473: 13. 2015

#### Gudeodiscus (Gudeodiscus) longiplica sp. n.

Taxon classificationAnimaliaPulmonataPlectopylidae

http://zoobank.org/3917B197-23F5-4274-B5AA-3B232691848A

Figures 2A–E , 3A–C , 4A–C , 5 , 7A–B , 8A , 9A–C , 10B

##### Material examined.

Guangxi (广西), Tiane Xian (天峨县), Liupai Zhen (六排鎮), Shuiliandong (水帘洞), 354 m, 25°00.623'N, 107°09.994'E, leg. Ishibe, T., Ohara, K., Okubo, K. & Otani, J. U., 21.10.2011, NHMUK 20150375 (holotype = shell + body in ethanol + radula on double-faced adhesive tape), NHMUK 20150376/1 paratype (= shell + body in ethanol), JUO/2 paratypes (= shells), PGB/1 paratype (= shell, ex coll. J.U. Otani); Same locality and collection data, OK/4 (= corroded paratypes; these four shells are paratypes of *Gudeodiscus soosi* as well).

##### Description of the shell

(Figs 2–4). The description is based on the holotype and a paratype (NHMUK 20150376) which was opened in order to examine the parietal plicae.

**Figure 1.**
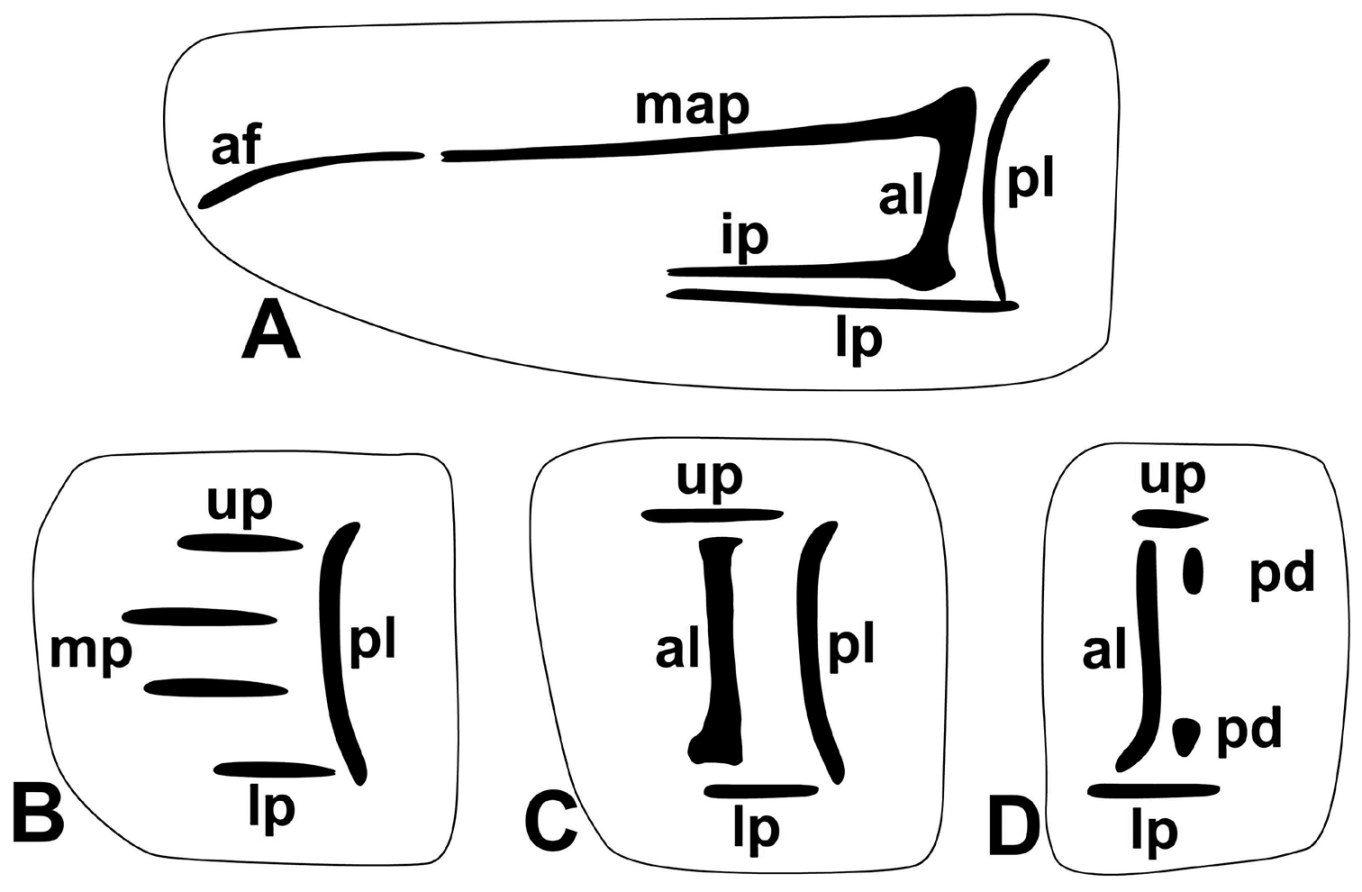
Nomenclature of the parietal plicae and lamellae of *Gudeodiscus* Páll-Gergely, 2013 (**A–C**) and *Endothyrella* Zilch, 1960 (**D**). **A** *Gudeodiscus longiplica* sp. n. **B** *Gudeodiscus emigrans quadrilamellatus* Páll-Gergely, 2013 **C** *Gudeodiscus phlyarius* (Mabille, 1887) **D** *Endothyrella* sp. Abbreviations: af: apertural fold; al: anterior lamella; ip: intermediate plica; lp: lower plica; map: main plica; mp: middle plicae; pd: posterior denticle; pl: posterior lamella; up: upper plica. In order to make the comparison easier, all figures show a dextral specimen (**D** is reversed). After Páll-Gergely and Hunyadi (2013) and Páll-Gergely et al. (2015b).

**Figure 2.**
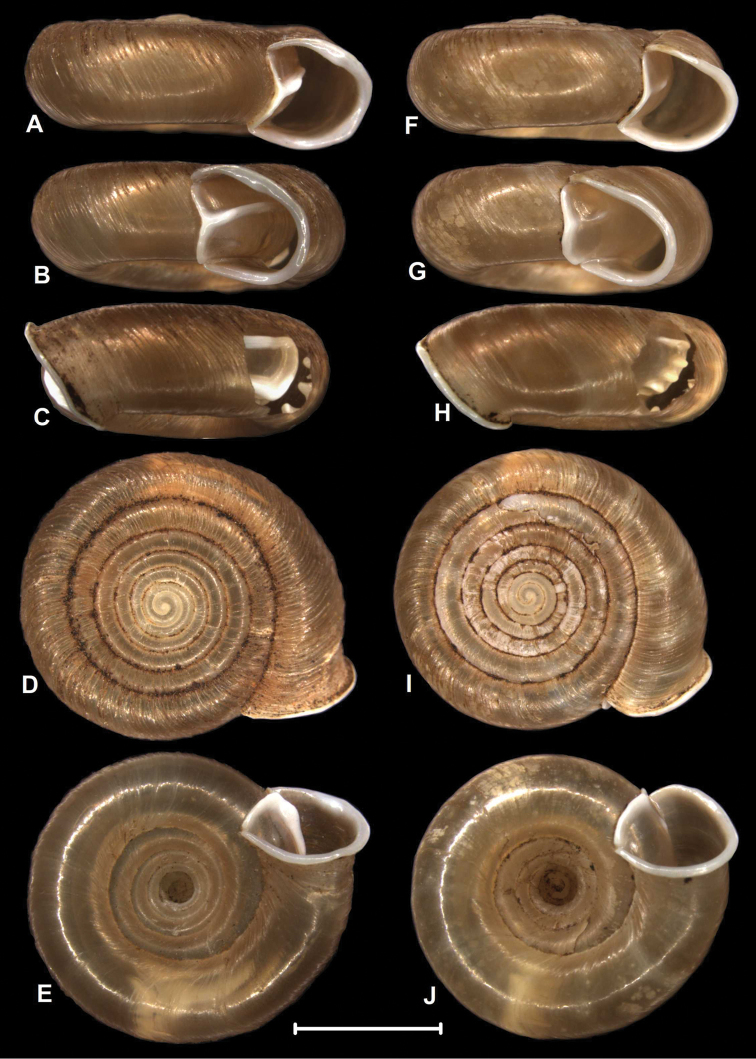
Shells of sympatric Chinese *Gudeodiscus* Páll-Gergely, 2013 species. **A–E** holotype of Gudeodiscus (Gudeodiscus) longiplica sp. n. **F–J** Gudeodiscus (Gudeodiscus) soosi Páll-Gergely, 2013, shell from the type locality (coll. JUO). Figures **C** and **H** were taken after they have been opened in order to observe the inner plicae. Scale bar: 5 mm.

**Figure 3.**
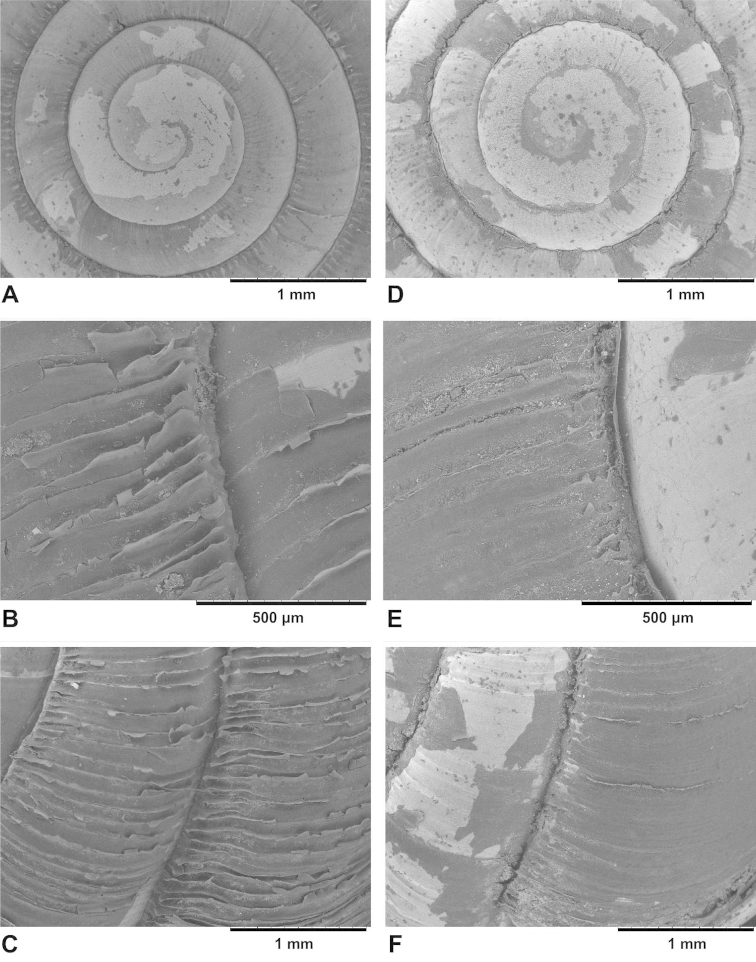
Protoconch (**A, D**) and teleoconch sculpture (**B–C, E–F**) of sympatric Chinese *Gudeodiscus* Páll-Gergely, 2013 species. **A–C** holotype of Gudeodiscus (Gudeodiscus) longiplica sp. n. **D–F**Gudeodiscus (Gudeodiscus) soosi Páll-Gergely, 2013, shell from the type locality (coll. JUO) **B** and **E** shows the last and penultimate whorls opposite of the aperture **C** and **F** shows the last and penultimate whorls near the aperture.

**Figure 4.**
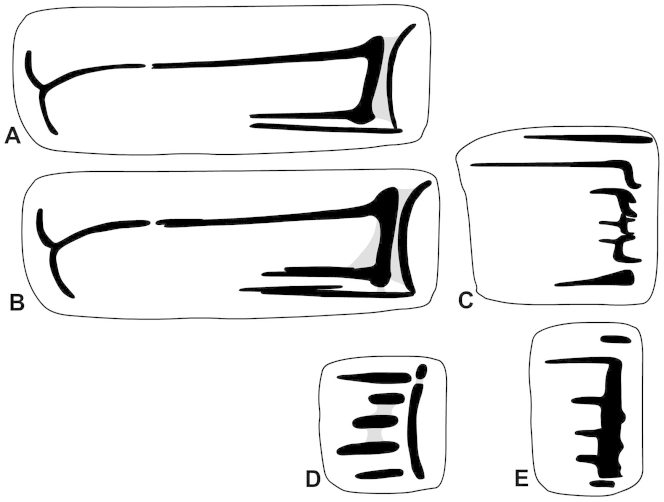
Parietal (**A, B, D**) and palatal plication of *Gudeodiscus* Páll-Gergely, 2013 species. **A** holotype of Gudeodiscus (Gudeodiscus) longiplica sp. n. **B–C** paratype of Gudeodiscus (Gudeodiscus) longiplica sp. n. (NHMUK 20150376) **D–E** Gudeodiscus (Gudeodiscus) soosi Páll-Gergely, 2013 (coll. JUO).

Shell small, dextral, corneous-light brown, translucent, nearly flat, only the protoconch is elevated; whorls 6.75; suture shallow at the protoconch but very deep, even groove-like, near the aperture; protoconch lighter in colour than the rest of the shell, 2 whorls; its surface very finely granulated, matt, and rather regularly ribbed near the suture, ribs becoming weak anteriorly; radial sculpture weakest on the protoconch and becoming stronger towards the end of the protoconch; the umbilical side of the protoconch is not ribbed, but finely granulated, matt; from dorsal view the first three whorls of the teleoconch are irregularly wrinkled, glossy, and lighter coloured than the rest of the shell; this sculpture changes gradually (after approx. 2.5 whorls) to a more strongly ribbed, somewhat darker, less glossy surface which possesses fine periostracal filaments on the ribs; these radial periostracal filaments are most prominent near the suture; the ribbed dorsal surface gradually changes to a smooth, glossy surface at the edge of the body whorl; umbilicus wide, funnel-shaped, shows all whorls; aperture slightly oblique to the shell axis, peristome white, moderately thickened and very much reflexed; parietal callus strong, elevated but rather blunt; it is angled when it joins the apertural fold; apertural fold long, but free from the main plica.

Parietal wall with two lamellae; the anterior is very much elevated, rather straight but oblique to the shell axis; its lower end is situated more anteriorly than the upper end; the posterior lamella is much weaker (lower) than the anterior, it is C-shaped; the two separate lamellae are well distinguishable, but they are connected to each other by a white calcareous layer; main horizontal plica long, it is connected to the upper end of the anterior lamella; the main plica almost reaches the apertural fold, but in both examined specimens the two structures are free from each other; lower plica long, starts from the lower end of the posterior lamella and ends before the ending point of the main plica; lower plica free from the anterior lamella in case of the holotype, but in the paratype there is a weak connection between them; middle plica strong, starts from the lowermost point of the anterior lamella, and ends in the same position as the lower plica. In case of the paratype there are some additional short plicae in contact with the other, above mentioned plicae, namely: one above the anterior end of the main plica, one above the middle plica, and one above the anterior end of the lower plica. Palatal wall with six plicae; the first is long, slender, it is situated near the suture; the second is situated in comparatively large distance from the first; the second plica is even longer than the first, its posterior part is curved downwards; the last (6th) plica is relatively short, with pointed anterior and blunt posterior ends; the middle plicae (3rd to 5th) are complicated, with a shape similar to curly brackets when looking through the semi-transparent shell wall; the anterior leg of the “curly brackets” are longer than the posterior ones; when observing from inside, the middle plicae have a triangular, pointed tip.

##### Measurements

(in mm). D = 11.7, H = 4.6 (holotype); D = 10.3–12.2, H = 4.3–5.0 (paratypes, n = 5).

##### Characters of the genital structure

**Figure 5.**
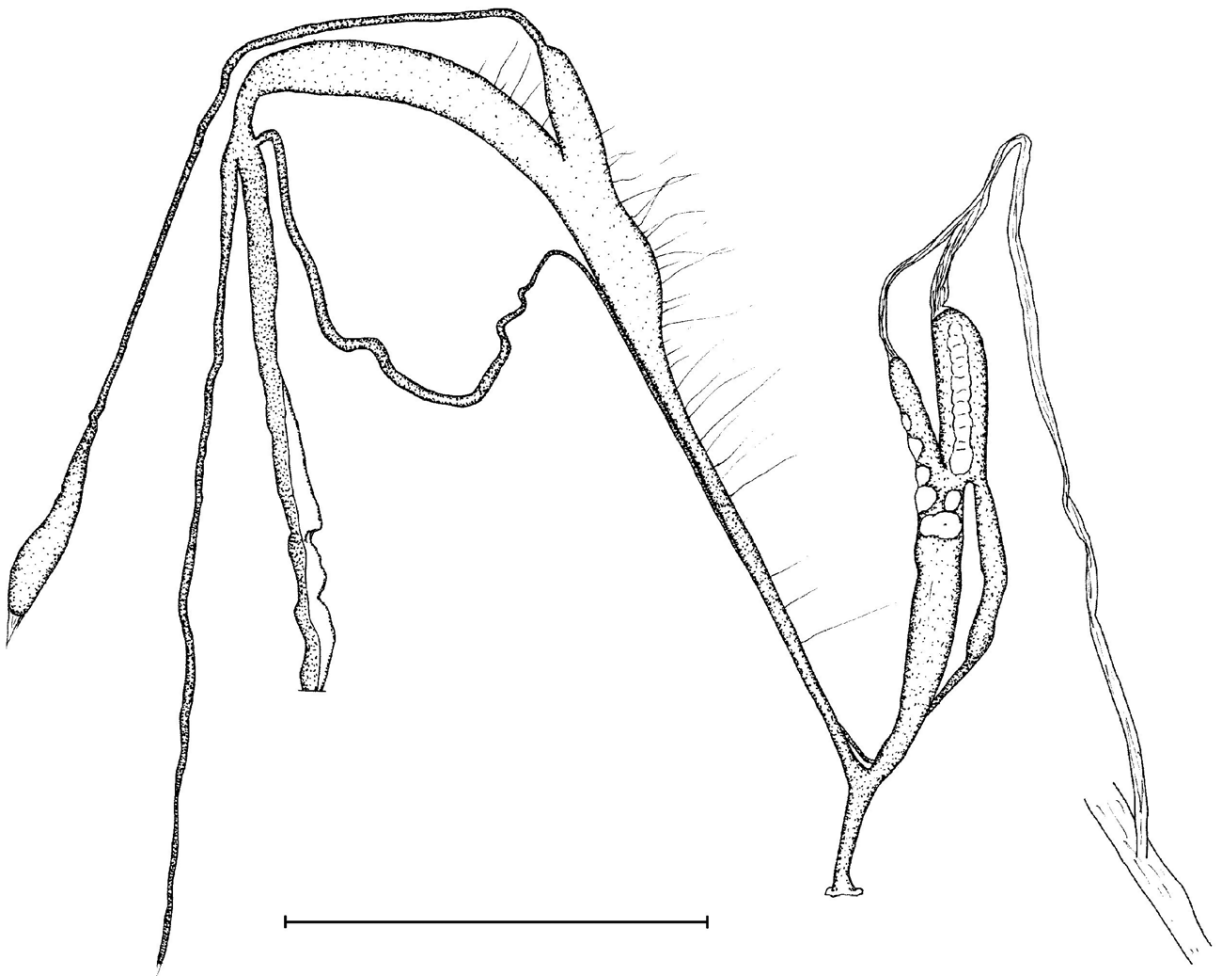
Genital anatomy of Gudeodiscus (Gudeodiscus) longiplica sp. n. Scale bar: 5 mm.

(Figs 5, 7A–B, 8A). Two specimens were anatomically examined. The right ommatophoral retractor passes between the penis and the vagina. Atrium slender, long; penis moderately long, with slimmer distal part; there are two penial caeca, both of them with their own retractor muscle which merge to a single fascicle after some distance; retractor muscle very long, branched off from the columellar muscle; one of the penial caeca is larger and slimmer than the other; the larger one is approximately half of the length of the penis; epiphallus enters penis laterally (at the meeting point of the penis and the larger penial caecum); penis internally with approximately 16 low, longitudinal folds; just distally from the joint of the epiphallus there is a single, straight, transversal row of small “pockets” which are formed by the longitudinal folds; we have not found calcareous objects in these pockets; larger penial caecum internally with a longitudinal, main row of rounded papillae; this main row consists of 8–9 papillae; there are other, smaller papillae arranged in 2–3 longitudinal rows on the inner wall; there are a few additional papillae adjacent to the ones of the main row; we have found a single calcareous granule with pointed tip and rounded, widened base in one of the papillae; the smaller penial caecum has a single, longitudinal row of papillae with approx. five papillae; the papillae are very well visible throughout the semi-transparent wall of the caeca; epiphallus as long as the longer penial caecum, internally with three longitudinal folds; vas deferens enters epiphallus apically, it is very slender, but gets thicker near the proximal portion of the vagina; vagina extremely long, approximately two times longer than the penis and the penial caecum combined; the distal part is very slender; the proximal portion is slightly thicker than the penis; the inner wall of the vagina is with irregular, low, longitudinal folds, which are the strongest at the proximal end of the vagina (closer to the joining point with the vas deferens); the bursa copulatrix starts a bit distally than the middle point of the proximal portion of the vagina; its base is thickened, but gets slimmer after a short distance; the stalk is slender and very long, the bursa is gradually thickened at the end; diverticulum starts at the end of the vagina, therefore the base of the diverticulum and the base of the bursa copulatrix are situated very far from each other; diverticulum very slender, without thickening at its end; it is approximately as long as the bursa copulatrix; a long, slender, glass-like, fragile spermatophore have been found in the diverticulum; spermoviduct very slender, long.

**Figure 6.**
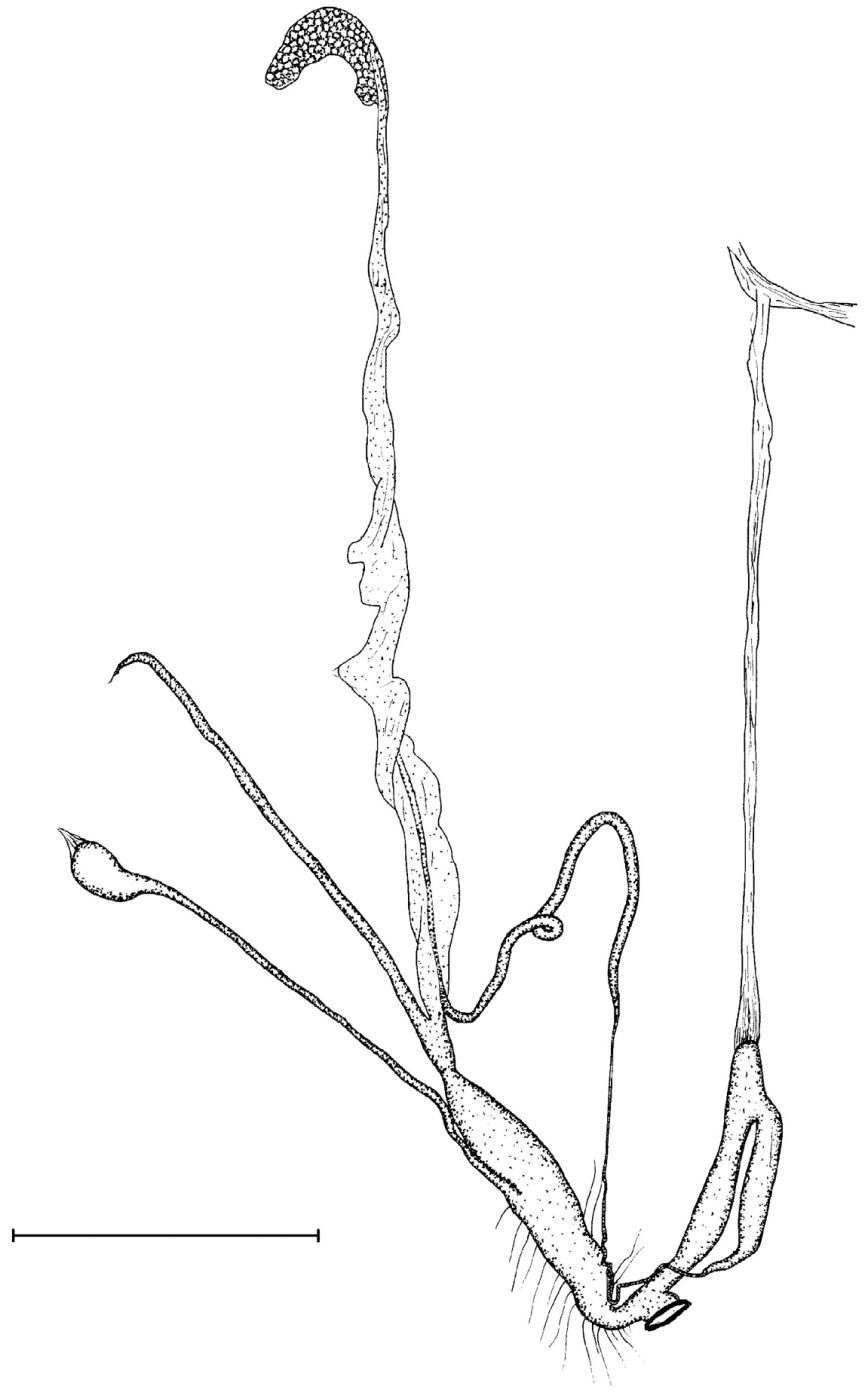
Genital anatomy of Gudeodiscus (Gudeodiscus) soosi Páll-Gergely, 2013. Scale bar: 5 mm.

**Figure 7.**
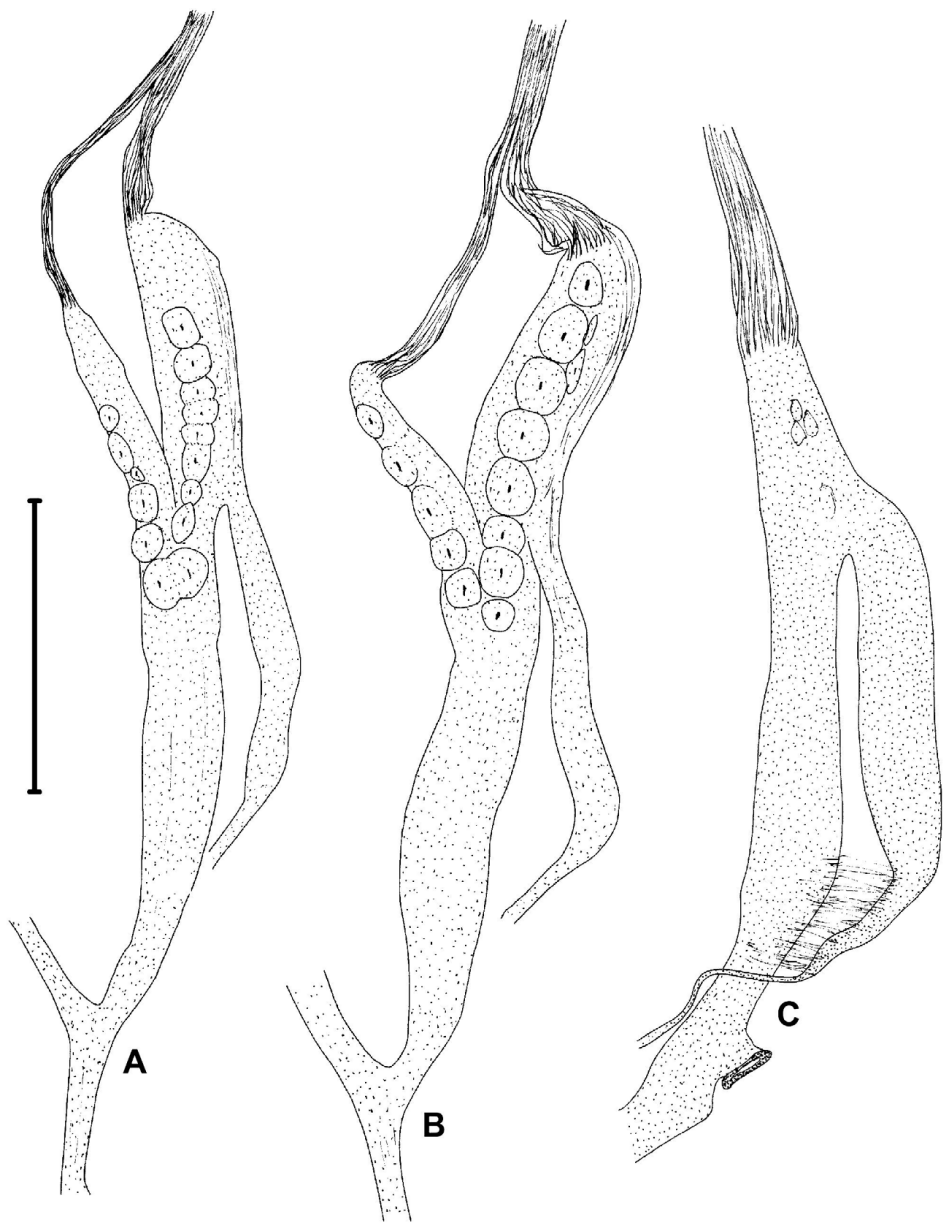
Male genitalia of Gudeodiscus (Gudeodiscus) longiplica sp. n. (**A, B**) and Gudeodiscus (Gudeodiscus) soosi Páll-Gergely, 2013 (**C**). Scale bar: 2 mm.

**Figure 8.**
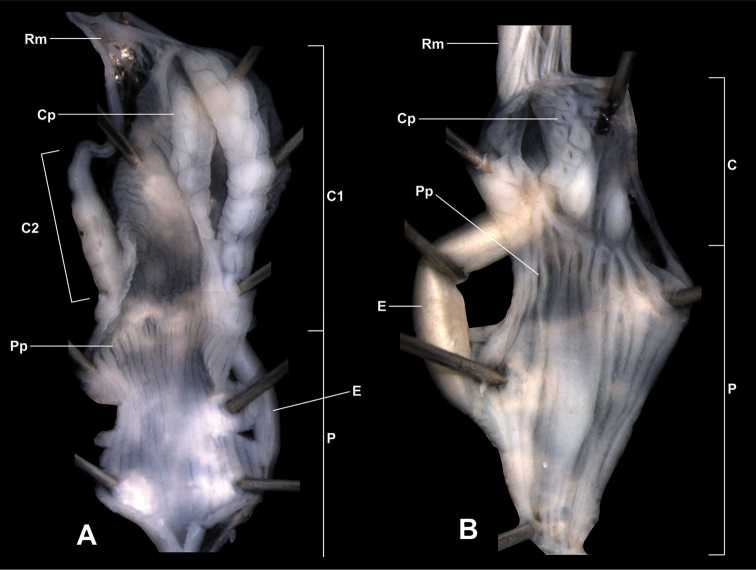
Opened penis and larger caecum of Gudeodiscus (Gudeodiscus) longiplica sp. n. (**A**) and Gudeodiscus (Gudeodiscus) soosi Páll-Gergely, 2013 (**B**). Abbreviations: C: penial caecum; C1: larger penial caecum; C2: smaller penial caecum; Cp: papilla on the inner wall of the larger penial caecum; E: epiphallus; P: penis; Pp: pockets on the penis wall; Rm: retractor muscle. Note that the most proximal portion of the penis is not shown on the left figure.

##### Characters of the radula

**Figure 9.**
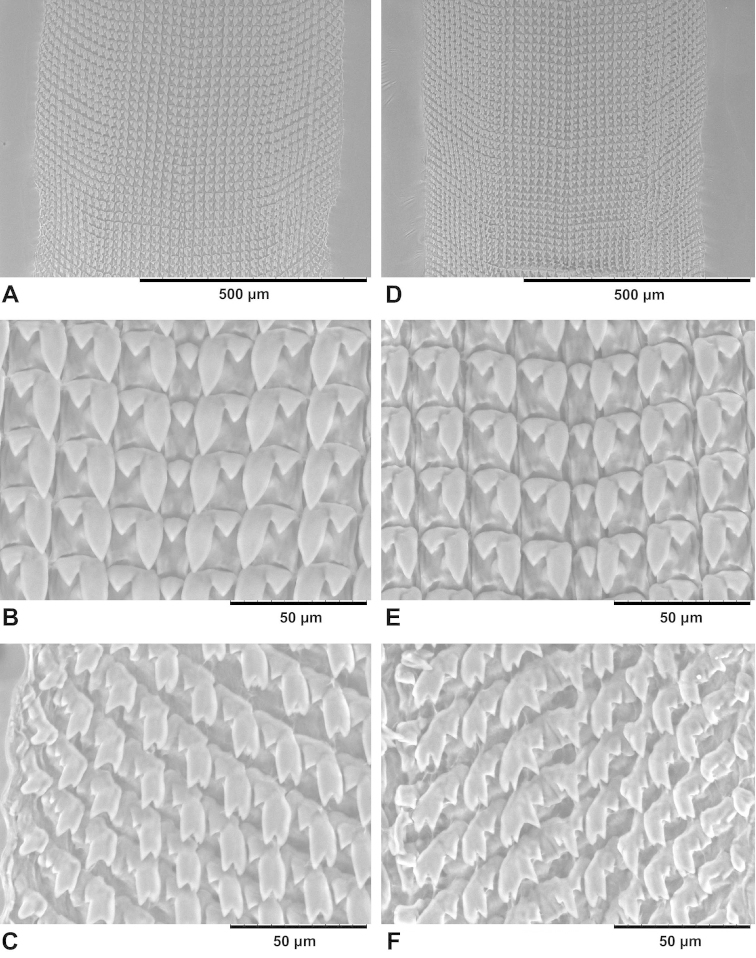
Radula of *Gudeodiscus* species. **A–C** Gudeodiscus (Gudeodiscus) longiplica sp. n. **D–F** Gudeodiscus (Gudeodiscus) soosi Páll-Gergely, 2013. **A, D** middle section of the radula plate **B, E** central tooth and first 3–4 lateral teeth **C, F** marginals.

(Fig. 9A–C). Radula elongated, but not very slender, central tooth present, laterals 7 or 8 (it is difficult to decide whether the 8th row belong to the laterals or the marginals), standing in straight lines (perpendicular to the central column); marginals approximately 11–12; marginals are placed in slightly oblique rows; central tooth wide-based triangular, smaller than the endocone of the first lateral, but approximately as large as the ectocone of the first laterals; laterals bicuspid, ectocones triangular, endocones have rather parallel margins with triangular, pointed tip; marginals usually tricuspid (= the endocone has two cusps); occasionally the innermost cusp is also divided into two cusps resulting in three cusps of the structure equivalent to the endocone of the laterals; some of the external marginals have both the endocone and the ectocone divided into two cusps; all cusps pointed, the incision between the innermost two cusps (= two cusps of the endocone) is deep.

##### Differential diagnosis.

*Gudeodiscus longiplica* sp. n. differs from all other *Gudeodiscus* species by the morphology of the parietal plicae and lamellae, and the presence of two penial caeca. It differs from the sympatric *Gudeodiscus soosi* by the presence of two well-developed parietal lamellae and three horizontal plicae (main, lower, and middle), as well as the apertural fold (*longiplica* sp. n.: long; *soosi*: short), the palatal plicae (*longiplica* sp. n.: first two long; *soosi*: first very short, second moderately long), the shell shape (*longiplica* sp. n.: dorsal side flat; *soosi*: dorsal side slightly domed) and the fine sculpture of the dorsal side (*longiplica* sp. n.: several radial periostracal folds; *soosi*: nearly smooth).

The long parietal plicae of *Gudeodiscus longiplica* sp. n. is similar to those of some, mostly sinistral species of *Chersaecia* and *Plectopylis*, which inhabit north-eastern India, Myanmar, northern Thailand, and northern Malaysia. The anatomy of *Plectopylis* and *Chersaecia* is insufficiently known, therefore we cannot use the anatomical characters of *Gudeodiscus longiplica* sp. n. to reject a close relationship with *Plectopylis* and *Chersaecia*. *Gudeodiscus longiplica* sp. n. has a regularly ribbed protoconch, whereas *Plectopylis* and *Chersaecia* species have finely tuberculated or smooth embryonic whorls (Schileyko 1999, Páll-Gergely et al. 2015b). Moreover, the palatal plicae of *Chersaecia* and *Plectopylis* are different (see Discussion).

##### Etymology.

This new species is named for its long plicae on the parietal wall.

##### Type locality.

Guangxi (广西), Tiane Xian (天峨县), Liupai Zhen (六排鎮), Shuiliandong (水帘洞), 354 m, 25°00.623'N, 107°09.994'E.

##### Distribution.

*Gudeodiscus longiplica* sp. n. is known only from the type locality.

#### Gudeodiscus (Gudeodiscus) soosi

Taxon classificationAnimaliaPulmonataPlectopylidae

Páll-Gergely, 2013

Figures 2F–J , 3D–F , 4D–E , 6 , 7C , 8B , 9D–F

Gudeodiscus soosi Páll-Gergely, In: Páll-Gergely & Hunyadi, Archiv für Molluskenkunde 142(1): 31–32, figs 42a–b, 66. 2013

##### Characters of the genital structure

(Figs 6, 7c, 8B). Two specimens were anatomically examined. Shells, ethanol-preserved bodies and radulae on double-faced adhesive tape are deposited in coll. JUO.

One of the specimens was aphallic, i.e. the male part of the genitalia was entirely missing. The right ommatophoral retractor passes between the penis and the vagina of the second specimen. Atrium extremely short, slender, long; penis moderately long, spindle shaped; inner wall of the penis with approx. 14 low longitudinal folds which join each other in the direction of the atrium resulting in fewer number of folds posteriorly; on the penial wall of the apical part of the penis there are slit-like “pockets” arranged in a transversal row; no calcareous granules have been found inside these pockets; the penial caecum is situated on the apical portion of the penis, it is approx. one third of the length of the penis; its inner wall is ornamented with several rhomboid papillae with holes in the middle of each papillae; no calcareous granules were found in them; retractor muscle inserts on the apical part of the penial caecum; retractor muscle very long, branched off from the columellar muscle; epiphallus enters penis laterally, at the joint of the penis and the larger penial caecum; its inner wall with three strong longitudinal folds; vagina slightly longer and thicker than the penis; the inner wall of the vagina is with irregular, low, longitudinal folds; the bursa copulatrix starts on the proximal part of the vagina; its base is not thickened; the stalk is slender and very long, the bursa is oval, more thickened than in the other species; diverticulum starts at the end of the vagina, therefore the base of the diverticulum and the base of the bursa copulatrix are very far from each other; diverticulum very slender, without thickening at it end; it is approximately as long as the bursa copulatrix; spermoviduct contained several developing eggs.

##### Characters of the radula

(Fig. 9D–E). Radula elongated, but not very slender, central tooth present, laterals 7 or 8 (it is difficult to decide whether the 8^th^ row belong to the laterals or the marginals), standing in straight lines (perpendicular to the central column); marginals approximately 12–13; marginals are placed in slightly oblique rows; central tooth wide-based triangular, smaller than the endocone of the first lateral, but approximately as large as the ectocone; laterals bicuspid, ectocones triangular, endocones have rather parallel margins with triangular tip; marginals usually tricuspid (= the endocone has two cusps); occasionally the innermost cusp is also divided into two cusps resulting in three cusps for the structure equivalent to the endocone of the laterals; some of the external marginals have both the endocone and the ectocone divided into two cusps; all cusps pointed, the incision between the innermost two cusps (= two cusps of the endocone) is deep.

##### Differential diagnosis.

See under *Gudeodiscus longiplica* sp. n.

##### Remarks.

We cannot rule out the possibility that the aphallic individual was a hybrid (see Schilthuizen et al. 2011).

## Discussion

*Gudeodiscus soosi* and *Gudeodiscus longiplica* sp. n. share an anatomical character that differentiate them from all other anatomically-known species of the Plectopylidae, including *Gudeodiscus*. The origination sites of the bursa copulatrix and the diverticulum are distantly situated from each other because the bursa copulatrix of both species branches off the vagina at approximately the middle vaginal section. In contrast, in all other members of Plectopylidae the bursa copulatrix and the diverticulum originate very near each other with both attaching at the proximal end of the vagina. This, however, does not warrant a genus-group-level distinction of *Gudeodiscus longiplica* sp. n. and *Gudeodiscus soosi* from other plectopylids. The general shell characters and the inner morphology of the penis (presence of a transversal row of slit-like “pockets”) places these two species in the genus *Gudeodiscus*. Furthermore, the retractor muscle inserts at the end of the penial caecum without additional curtain-like muscle fibres (characteristic for the subgenus *Veludiscus* Páll-Gergely, 2015) on the apical part of the penis. This trait places *Gudeodiscus longiplica* sp. n. and *Gudeodiscus soosi* in the subgenus Gudeodiscus (Gudeodiscus). The morphology of radular teeth also agrees with the other members of the subgenus *Gudeodiscus*. Namely, the central tooth is as large as the ectocone of the first laterals, the marginals are tricuspid or even quadricuspid with rather pointed inner cusp, and there is a deep incision between the two inner cusps. In contrast, Gudeodiscus (Veludiscus) species are characterized by central teeth smaller than the ectocone of the first laterals, and the inner cusps of the marginals are rather blunt with shallow incision between the two innermost cusps.

*Gudeodiscus longiplica* sp. n. has two surprising characters that need further discussion. Firstly, the two long, anteriorly-elongated parietal plicae that are in contact with the anterior lamellae, and secondly, its two separate penial caeca, which are similar in inner and outer morphology, but differ in size.

### Long parietal plicae

**Figure 10.**
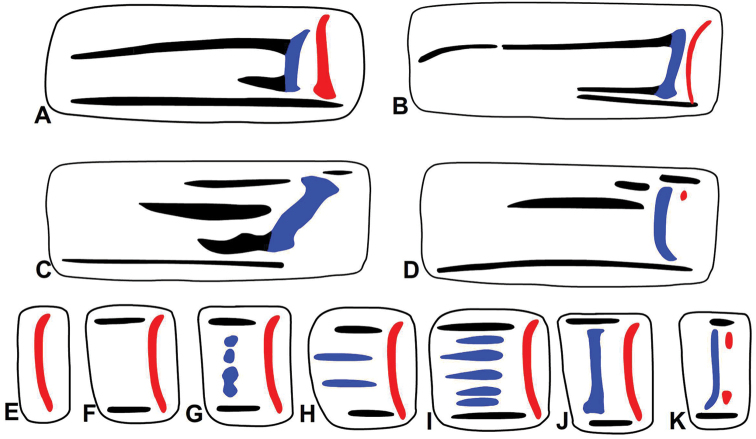
Parietal plication of Plectopylidae species (diagrammatic figures). **A** *Plectopylis leucochila* Gude, 1898 (after Gude 1898) **B** Gudeodiscus (Gudeodiscus) longiplica sp. n. **C** *Endothyrella brahma* (Godwin-Austen, 1879) **D** *Endothyrella williamsoni* (Gude, 1915) **E–J** character states of *Gudeodiscus*, *Halongella*, *Sicradiscus* and *Sinicola*; **K** *Endothyrella* sp. (some *Sicradiscus* has also similar parietal lamellation) (mainly after Páll-Gergely & Hunyadi 2013 and Páll-Gergely et al. 2015b). To allow better comparison all figures show dextral specimens (**A, C, D**, **J** are reversed), thus, the aperture is situated left from the armature. Red colour indicates the posterior, blue colour indicates the anterior lamella (and their respective homologous structures).

*Gudeodiscus*, *Halongella* Páll-Gergely, 2015, *Sicradiscus* Páll-Gergely, 2013 and *Sinicola* Gude, 1899 are known as genera lacking long horizontal parietal plicae on the parietal wall. Species belonging to these four genera possess two vertical lamellae (Fig. 10J), a single lamella (Fig. 10E–F), or a single lamella with denticles anteriorly, which are situated in the position of the anterior lamella (Fig. 10G). In some *Sicradiscus* and in most *Endothyrella* Zilch, 1960 species there is a single lamella and one or two denticles on its posterior side (Fig. 10K). These posterior denticles are probably homologous with the posterior lamella. Only some taxa, namely two subspecies of *Gudeodiscus emigrans* (Möllendorff, 1901) and *Sinicola reserata hensanensis* (Yen, 1939) are reported to have four relatively short, anteriorly elongated plicae (Fig. 10H). *Gudeodiscus ursula* Páll-Gergely, 2013 has seven parallel plicae, the uppermost and the lowermost being conspicuously longer and slimmer than the middle ones (Fig. 10I) (Páll-Gergely and Hunyadi 2013). The single, vertical, curved lamella of these three species is probably homologous with the posterior lamella of other *Gudeodiscus* species which possess two lamellae. The middle horizontal plicae anterior to the single lamella, however, are situated at the position of the anterior lamella. Thus, the two middle plicae are probably homologous with the anterior lamella. There are even transitional character states between the two lamella-type (Fig. 10J) and the single lamella plus four parallel plica-type (Fig. 10H) (see Páll-Gergely and Asami 2014: figures 5F–H). In *Gudeodiscus longiplica* sp. n., the anteriorly elongated plicae are connected to the well-developed anterior lamella (Fig. 10B). Therefore, the long horizontal plicae of *Gudeodiscus longiplica* sp. n., and those of the above-mentioned two *Gudeodiscus* and one *Sinicola* species cannot be homologous. Instead, the horizontal parietal plicae of *Gudeodiscus longiplica* sp. n. are probably homologous with those of the genera *Plectopylis* and *Chersaecia*, where long plicae commonly occur. Among the plectopylid genera possessing ribbed embryonic whorls (*Gudeodiscus*, *Sicradiscus*, *Sinicola*, *Halongella*, *Endothyrella*) only some *Endothyrella* species possess long horizontal lower and/or main parietal plicae (Fig. 10C–D) (Páll-Gergely et al. 2015b). The long plicae are probably plesiomorphic characters in the Plectopylidae.

### Double penial caecum

Both anatomically examined specimens of *Gudeodiscus longiplica* sp. n. had two separate penial caeca, both having their own fascicle of retractor muscle. The two caeca were different in length but the outer appearance and the inner structure were similar. The same anatomical trait in both specimens suggests that the two caeca are characteristic for the species and do not represent rare teratological event. No other members of the Plectopylidae are known to have two separate penial caeca. Moreover, as our non-exhaustive literature survey shows, the duplication of the penial caecum (or any accessory organ in similar relative positions) is a very rare event in stylommatophoran snails.

Two genera of the Cerastidae (= Pachnodidae; superfamily Enoidea), namely *Altenaia* Zilch, 1972 and *Archeorachis* Schileyko, 1998 possess two penial caeca arising from the apical part of the penis. None of these caeca have retractor muscles. One caeca is slender, vermiform, and the other is conic or ovate. Other genera of the Pachnodidae are known to possess only one type of the penial caeca, either vermiform or thick and fleshy (Schileyko 1998). The euconulid genus *Gunongia* Tillier & Bouchet, 1988 and the systrophiid *Tamayoa* Baker, 1925 (see Tillier 1980) also possess two differently looking penial caeca without retractor muscles. Some species of the genus *Deroceras* Rafinesque, 1820 (family Agriolimacidae) have two accessory organs on the apical part of the penis (Wiktor 2000). These organs differ in morphology and function from each other (“penial caecum” and “penial lobe”, see Reise et al. 2011, H. Reise & J. Hutchinson, pers. comm., 2015). These accessory organs usually lack retractor muscles, but *Deroceras oertzeni* (Simroth, 1889) has a branched penis retractor running to more or less the ends of two big pockets (bigger one is considered the main penis and the other one an “appendix”, see Wiktor 2001). The different morphology of the two caeca of the Pachnodidae, Euconulidae, Systrophiidae and Agriolimacidae are not the result of the duplication of a single organ, as we hypothesise in the case of *Gudeodiscus longiplica* sp. n. Moreover, retractor muscles of the penial appendices are absent in most above-mentioned taxa, but present in *Gudeodiscus longiplica* sp. n.

In the literature we encountered some reports of retractor muscles with two branches, each of them inserting on the two penial caeca, or the penial caecum and the penis itself. *Furcopenis darioi* Castillejo & Wiktor, 1983, (Agriolimacidae) has a bifurcate “accessory organ” with retractor muscles inserting on both tips of the accessory body (Castillejo and Wiktor 1983, Wiktor 2000). *Testacella scutulum* G.B. Sowerby I, 1820 and species of the genus *Schistophallus* Wagner, 1915 have the two branches of the retractor muscle inserted on the bifurcated end of the penis (Wagner 1915; *Testacella scutulum* was mentioned under the name *Testacella hungarica*). Despite the superficial similarity between the above mentioned pairs of penial accessory structures and the doubled penial caecum of *Gudeodiscus longiplica* sp. n., it is difficult to decide whether these organ pairs represent homologous structures between distantly related taxonomic groups, especially since our knowledge of their function is extremely limited. The very similarly looking pair of penial caeca in *Gudeodiscus longiplica* sp. n. represents a rare and interesting case which requires further investigation.

In most *Gudeodiscus* species the penis has larger pockets for calcareous hook-like granules than the penial caecum. In some examples the caecum was absent (Páll-Gergely and Asami 2014, Páll-Gergely et al. 2015a). Assuming that the penial and caecal calcareous hooks have similar functions, this suggests that whatever function the hooks might have (stimulation, mucus injection, mechanical holdfast, see Páll-Gergely et al. 2015a), the penis makes a larger contribution than the penial caecum. The penial pockets of *Gudeodiscus longiplica* sp. n. and *Gudeodiscus soosi* are smaller and shallower than the caecal ones. We might assume that in the two species in question (but especially in *Gudeodiscus longiplica* sp. n.) the hooks in the penial caecum play a more important role during mating than the hooks in the penis.

## Supplementary Material

XML Treatment for Gudeodiscus

XML Treatment for Gudeodiscus

XML Treatment for Gudeodiscus (Gudeodiscus) longiplica

XML Treatment for Gudeodiscus (Gudeodiscus) soosi
